# Functional and Preliminary Characterisation of Hydrocolloid from Tamarillo (*Solanum betaceum* Cav.) Puree

**DOI:** 10.3390/molecules17066869

**Published:** 2012-06-05

**Authors:** Sri Puvanesvari Gannasin, Yogeshini Ramakrishnan, Noranizan Mohd. Adzahan, Kharidah Muhammad

**Affiliations:** Faculty of Food Science and Technology, Universiti Putra Malaysia, Serdang 43400, Selangor Darul Ehsan, Malaysia

**Keywords:** tamarillo, hydrocolloid, extraction, functional, FT-IR, RP-HPLC

## Abstract

Hydrocolloid from tamarillo (*Solanum betaceum* Cav.) puree was extracted using water and characterised for the first time. Proximate compositions of the extracted hydrocolloid were also determined. Functional characteristics such as water-holding capacity, oil-holding capacity, emulsifying activity, emulsion stability, foaming capacity and stability of the hydrocolloid were evaluated in comparison to that of commercial hydrocolloids. Its functional groups and degree of esterification were determined using Fourier Transform Infrared (FT-IR) spectroscopy. Monosaccharide profiling was done using reverse-phase high pressure liquid chromatography (RP-HPLC). Screening of various fruits for high hydrocolloid yield after water extraction resulted in tamarillo giving the highest yield. The yield on dry weight basis was 8.30%. The hydrocolloid constituted of 0.83% starch, 21.18% protein and 66.48% dietary fibre with 49.47% degree of esterification and the monosaccharides identified were mannose, ribose, rhamnose, galacturonic acid, glucose, galactose, xylose and arabinose. Higher oil-holding capacity, emulsifying activity and emulsion stability compared to commercial hydrocolloids propose its possible application as a food emulsifier and bile acid binder. Foaming capacity of 32.19% and good foam stabilisation (79.36% of initial foam volume after 2 h of foam formation) suggest its promising application in frothy beverages and other foam based food products. These findings suggest that water-extracted tamarillo hydrocolloid can be utilised as an alternative to low methoxyl pectin.

## 1. Introduction

Hydrocolloids refer to a range of polysaccharides and proteins that are widely used in the food, cosmetic and pharmaceutical industries. The food industry, in particular, has seen a large increase in the use of hydrocolloids in recent years to perform a number of functions, including thickening and gelling aqueous solutions, stabilising foams, emulsions and dispersions, inhibiting ice and sugar crystal formation and the controlled release of flavours [1,2,3]. This multitude of functions can be achieved by incorporating hydrocolloids at 1% concentration or less which can have a significant influence on the textural and organoleptic properties of food products [3,4].

Hydrocolloids which are high molecular weight hydrophilic biopolymers include polysaccharides from various sources such as seaweeds, microorganisms, plants and animals. In addition, the unique protein gelatine is also classified as a hydrocolloid [1,3,5].

The world hydrocolloid market which is valued around USD 4.4 billion p.a. with a total volume of about 260,000 tonnes in 2000 reflects a remarkable demand for hydrocolloids which inevitably influences the price and security of supply [3]. Parallel with the increase in the hydrocolloid market demand, growing research interest to exploit some new raw materials as potential sources of food hydrocolloids can be observed. Hydrocolloids extracted from plants especially, have an advantage over those from microorganisms and animals because of their friendly image towards consumers.

Since vegetables consist predominantly of insoluble fibre fraction, fruits are of much interest among researchers to study the soluble fibre fraction which has good technological and health functionalities [6,7,8,9,10]. Preliminary comparative study of various tropical and sub-tropical fruits using the water extraction method indicated that tamarillo (*Solanum betaceum* Cav.), an undervalued fruit in Malaysia, gave the highest hydrocolloid yield.

Tamarillo or tree tomato is a subtropical fruit native to the Andes region of Peru, Chile, Ecuador, and Bolivia. It is also being cultivated in other countries, such as Argentina, Australia, Brazil, Colombia, Indonesia, Kenya, Malaysia (Cameron Highlands), New Zealand, Portugal and Venezuela [11,12,13]. Tamarillo types are best distinguished according to their peel and pulp colours. The type which is available in Malaysia is the egg-shaped, with thin reddish-brown skin and orange pulp, and dark red seed mucilage coating the seeds. To date, this cheap (USD 1/kg in Malaysia) and underutilised fruit remains unexplored except for its antioxidant profile [12,14,15]. Hence, this study is dedicated to investigate the possible exploitation of tamarillo as a food hydrocolloid source. Being extracted for the first time, important functional characteristics such as water-holding and oil-holding capacities, emulsifying activity, emulsion stability, foaming capacity and stability of the tamarillo hydrocolloid were studied in comparison to that of commercial hydrocolloids. In addition, the functional groups present in the tamarillo hydrocolloid was identified using Fourier Transform Infrared (FT-IR) spectroscopy. The monosaccharide composition of tamarillo hydrocolloid was also studied using reverse-phase high pressure liquid chromatography (RP-HPLC). These findings were expected to provide a basis for better understanding of this newly extracted hydrocolloid that might have a great importance in various food applications.

## 2. Results and Discussion

### 2.1. Hydrocolloid Yield of Various Tropical and Sub-Tropical Fruits Using Water Extraction Method

Screening of 15 fruits for high hydrocolloid yield (Y, % dry weight) revealed that the tamarillo pulp fraction had the highest hydrocolloid content (8.39%), followed by tamarillo puree (8.30%), papaya (7.23%), mango (6.62%) and sapodilla (5.66%) pulps, as shown in Table 1.

**Table 1:** Hydrocolloid yield (% fresh and dry weight) of various tropical and sub-tropical fruits.

Common name	Botanical name	Fraction	Moisture content (%)	Yield, Y_f_ (% fresh weight)	Yield, Y (% dry weight)
Tamarillo (buah cinta)	*Solanum betaceum* Cav.	Pulp	85.82 ^a^ ± 0.13	1.19 ^a^ ± 0.02	8.39 ^a^ ± 0.03
		Seed mucilage	88.70 ^b^ ± 0.30	0.40 ^bf^ ± 0.08	3.54 ^b^ ± 0.16
		Puree	85.78 ^a^ ± 0.09	1.18 ^ac^ ± 0.10	8.30 ^c^ ± 0.07
Papaya	*Carica papaya*	Pulp	84.64 ^c^ ± 0.04	1.11 ^acd^ ± 0.06	7.23 ^d^ ± 0.15
Sapodilla (ciku)	*Manilkara zapota*	Pulp	80.92 ^d^ ± 0.06	1.08 ^cd^ ± 0.11	5.66 ^e^ ± 0.23
Mango	*Mangifera indica*	Pulp	84.28 ^ce^ ± 0.03	1.04 ^d^ ± 0.13	6.62 ^f^ ± 0.07
Kiwifruit	*Actinidia deliciosa*	Pulp	87.01 ^f^ ± 0.04	0.90 ^e^ ± 0.04	6.93 ^g^ ± 0.15
Mandarin orange	*Citrus reticulata*	Peel	75.20 ^g^ ± 0.27	0.47 ^b^ ± 0.02	1.90 ^h^ ± 0.10
Garden tomato	*Lycopersicon esculentum*	Whole without seeds	91.24 ^h^ ± 0.15	0.42 ^bf^ ± 0.08	4.79 ^i^ ± 0.08
Pineapple	*Ananas comosus*	Pulp	89.95 ^i^ ± 0.03	0.40 ^bf^ ± 0.03	3.98 ^j^ ± 0.11
Marian plum (kundang)	*Bouea macrophylla*	Pulp	84.13 ^e^ ± 0.15	0.34 ^f^ ± 0.10	2.14 ^k^ ± 0.03
Red dragon fruit	*Hylocereus polyrhizus*	Pulp	87.69 ^j^ ± 0.08	0.33 ^f^ ± 0.11	2.68 ^l^ ± 0.07
Guava	*Psidium guajava*	Whole without seeds	91.29 ^h^ ± 0.08	0.20 ^g^ ± 0.02	2.30 ^m^ ± 0.06
Water apple (jambu air)	*Syzygium aqueum*	Whole	92.50 ^k^ ± 0.03	0.20 ^g^ ± 0.03	2.67 ^l^ ± 0.06
Jackfruit	*Artocarpus heterophyllus*	Pulp	77.92 ^l^ ± 0.33	0.2 0^g^ ± 0.05	0.91 ^n^ ± 0.09
Honeydew	*Cucumis melo*	Whole without seeds	94.36 ^m^ ± 0.03	0.20 ^g^ ± 0.02	3.55 ^b^ ± 0.23
Red apple	*Malus pumila*	Whole without seeds	83.56 ^n^ ± 0.23	0.16 ^g^ ± 0.06	0.97 ^o^ ± 0.04
Cupuassu	*Theobroma grandiflorum*	Pulp	NA	NA	7.00 ^g^ ± NA [16] *
Longan	*Dimocarpus longan*	Pulp	NA	NA	4.46 ^i^ ± 0.09 [17] *
Gold kiwifruit	*Actinidia chinensis*	Whole	80.99 ^d^ ± 0.14	NA	6.69 ^f^ ± NA [18] *

Each value is expressed as mean ± standard deviation (n = 3) of triplicate analysis. ^a–o^ Means followed by different superscripts indicate significant differences (*p* < 0.05) within column by Tukey’s test. * Published data.

**Figure 1:**
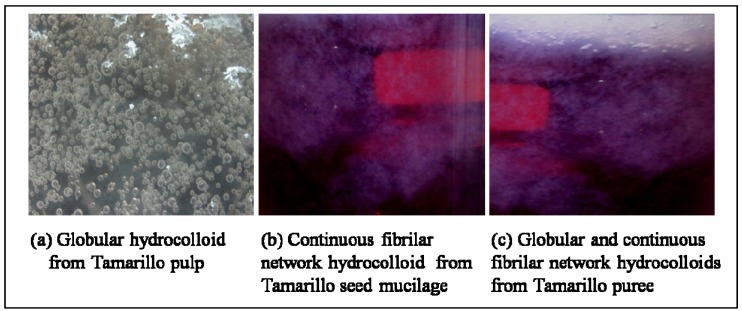
Visual presence of globular and continuous fibrilar network hydrocolloids (after precipitation in chilled aqueous ethanol) from tamarillo fruit fractions.

Hydrocolloids from these fruit fractions appeared in a globular shape (Figure 1a) when immersed in chilled aqueous ethanol, except for tamarillo puree. Another type of hydrocolloid which has a continuous fibrilar network (Figure 1b) was observed when extraction was done with tamarillo seed mucilage, kiwifruit, dragon fruit pulp, cored apple, orange peel and other fruits that were screened.

Figure 1 shows the visual presence of globular and continuous fibrilar network hydrocolloids that were observed during hydrocolloid extraction from tamarillo pulp, seed mucilage and puree. The differences between pulp, seed mucilage and puree fractions in relation to the morphological parts of tamarillo fruit were described in Section 3.2. It can be noted that tamarillo puree fraction has both globular and continuous fibrilar network hydrocolloids (Figure 1c). Since it is not feasible and economical for the food industry to extract either the pulp or seed mucilage hydrocolloid of the fruit, the puree fraction (combination of pulp and seed mucilage fractions) was studied. It is worth highlighting here that water extracted hydrocolloid from tamarillo puree (TH_water_) accounted for the highest yield compared to that of other fruits that were screened and previously studied [16,17,18] using water extraction method.

### 2.2. Proximate Compositions of Tamarillo Hydrocolloid

Table 2 shows the proximate compositions of TH_water_. It is interesting to note that the protein content was quite high (21.18 ± 0.06%) which might be contributed by the seed mucilage fraction in the tamarillo puree. A small amount of starch was also detected in TH_water_, besides the major component, dietary fibre, which accounted for 66.48 ± 0.52%. Since ~90% of TH_water_ was made up of protein, starch and dietary fibre, the term hydrocolloid was used instead of polysaccharide. Protein and starch were purposely not removed from the extract by enzymatic treatment since these two components might contribute to good functional characteristics that are needed for various food applications.

**Table 2:** Proximate compositions of tamarillo hydrocolloid extracted using water (TH_water_).

Component	Composition
Moisture (%)	10.65 ± 0.32
Dry matter (%)	89.35 ± 0.32
Ash (% dry weight)	0.80 ± 0.09
Protein (% dry weight)	21.18 ± 0.06
Starch (% dry weight)	0.83 ± 0.06
Dietary fibre by difference ^a^ (% dry weight)	66.48 ± 0.52

Each value is expressed as mean ± standard deviation (n = 3) of triplicate analysis; ^a^ Dietary fibre by difference = 100% − (Moisture + Ash + Protein + Starch)%.

### 2.3. Functional Properties of Tamarillo Hydrocolloid in Comparison to that of Commercial Hydrocolloids

To date, there are no published research articles showing a comparison between the functional properties of commercial hydrocolloids from various sources. Therefore, this work is expected to help the food manufacturers especially in selecting the right hydrocolloid to be incorporated into food for specific functionalities.

#### 2.3.1. Water-Holding Capacity (WHC) and Oil-holding Capacity (OHC)

As shown in Table 3, there is no significant difference between WHC of apple pectin and TH_water_ and likewise for OHC.

**Table 3:** Water holding capacity (WHC) and oil holding capacity (OHC) of Tamarillo and commercial hydrocolloids.

Type of hydrocolloid	WHC (g water/g dry sample)	OHC (g oil/g dry sample)
TH_water_	5.82 ^a^ ± 0.75	2.00 ^ab^ ± 0.07
Agar-agar	7.99 ^b^ ± 0.80	2.25 ^b^ ± 0.07
Apple pectin	6.71 ^ab^ ± 0.52	2.11 ^ab^ ± 0.17
Bovine gelatine	0.00 ^c^ ± 0.00	1.06 ^cfg^ ± 0.03
Carrageenan	28.21 ^d^ ± 0.92	1.31 ^dh^ ± 0.05
Citrus pectin	1.38 ^c^ ± 0.06	1.55 ^de^ ± 0.09
CMC	0.00 ^c^ ± 0.00	1.58 ^e^ ± 0.03
Gum arabic	0.28 ^c^ ± 0.15	1.00 ^cf^ ± 0.10
Karaya gum	24.39 ^e^ ± 0.17	1.12 ^cfgh^ ± 0.02
Sodium alginate	0.00 ^c^ ± 0.00	1.22 ^fgh^ ± 0.02
Wheat starch	0.74 ^c^ ± 0.02	0.92 ^c^ ± 0.03
Xanthan gum	62.63 ^f^ ± 0.91	1.28 ^gh^ ± 0.03

Each value is expressed as mean ± standard deviation (n = 3) of triplicate analysis; ^a–h^ Means followed by different superscripts indicate significant differences (*p* < 0.05) within column by Tukey’s HSD test.

It can be observed that TH_water_ demonstrated higher WHC compared to that of bovine gelatine, citrus pectin, carboxymethyl cellulose sodium salt (CMC), gum arabic, sodium alginate and wheat starch. In contrast, the WHC of TH_water_ was lower than that presented by xanthan gum, carrageenan and karaya gum. These findings suggest that xanthan gum, carrageenan and karaya gum are better at reducing syneresis and modifying the texture of foods. In addition, hydrocolloids with high WHC can be used as fat replacers in low-fat/calorie food formulations [19,20]. The OHC of TH_water_ was higher than that shown by bovine gelatine, carrageenan, citrus pectin, CMC, gum arabic, karaya gum, sodium alginate, wheat starch and xanthan gum suggesting the application of TH_water_ as a food emulsifier.

#### 2.3.2. Emulsifying Activity (EA) and Emulsion Stability (ES)

**Figure 2:**
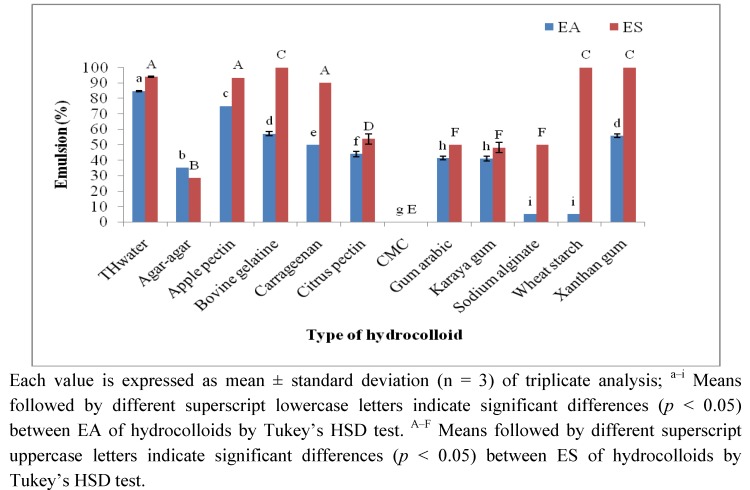
Emulsifying activity (EA) and emulsion stability (ES) of tamarillo and commercial hydrocolloids.

Figure 2 shows the EA and ES of tamarillo and commercial hydrocolloids. It is interesting to note that TH_water_ demonstrated the highest EA (84.74 ± 0.46%) compared to all the commercial hydrocolloids studied. This finding was consistent with the OHC of TH_water_ that confirmed the possible application of TH_water_ as a plant based food emulsifier in stabilisation of high fat food products and emulsions.

Moreover, the ES (94 ± 0.21%) of TH_water_ was higher than that of the widely used food emulsifier, gum arabic, which accounted for only 50 ± 0.02% of ES. Besides the technological functionality, high EA and ES of TH_water_ were also very indicative of its ability to adsorb biliary acids which is one of the main mechanisms to reduce cholesterol level in blood [21]. 

#### 2.3.3. Foaming Capacity (FC) and Foaming Stability (FS)

**Figure 3:**
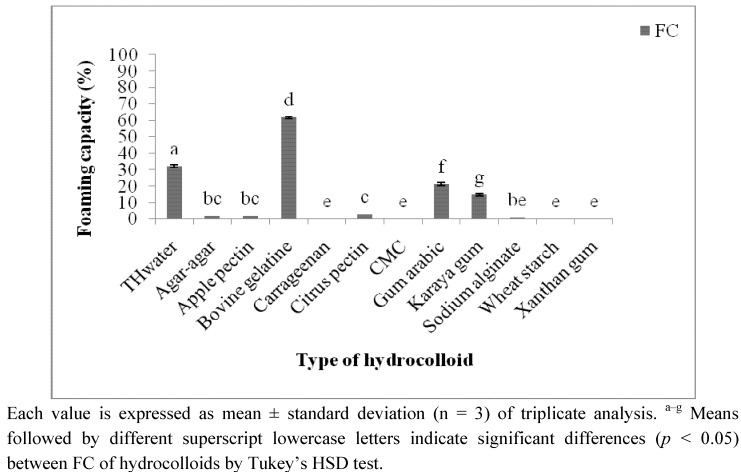
Foaming capacity (FC) of tamarillo and commercial hydrocolloids at time, t = 0.

Foam is a colloid system with a gaseous phase stabilised in a continuous matrix to provide an aerated structure to a food product [22]. As expected, bovine gelatine showed the highest FC (61.92 ± 0.68%), followed by TH_water_ that presented 32.19 ± 0.76% of FC (Figure 3).

Although the FC of TH_water_ was lower than that of (animal based) bovine gelatine, TH_water_ possessed a considerably high FC compared to other commercial hydrocolloids. This foaming characteristic of TH_water_ might be also be contributed by the protein present in the extract besides the major component, polysaccharides. Conversely, carrageenan, CMC, wheat starch and xanthan have no FC. Agar-agar, sodium alginate, apple and citrus pectin showed a very low FC, in the range of 1 to 3% only. 

Even though the FC of bovine gelatine was the highest, its FS reduced drastically from 90.36% (1 min after foam formation) to 11.01% (2 h after foam formation, Table 4). In contrast, TH_water_ successfully maintained the foam volume through the 2 h (79.36% of initial volume) suggesting its application as foam stabiliser in frothy beverages such as cappuccino and beer. It can also be utilised in foam based food products such as mousses, marshmallows and meringues.

Karaya gum, apple and citrus pectin showed intermediate FS of 48.73%, 50% and 66.67%, respectively after 2 h of foam formation. Gum arabic and sodium alginate showed a poorer FS. FS of carrageenan, CMC, wheat starch and xanthan gum were not determined since these hydrocolloids have no FC.

**Table 4:** Foaming stability (%) of tamarillo and commercial hydrocolloids.

Hydrocolloids	Foaming stability (%) at time, t (min)					
	t = 1	t = 10	t = 30	t = 60	t = 90	t = 120
TH_water_	97.59 ^aA^	91.96 ^aB^	84.99 ^aC^	81.51 ^aD^	79.36 ^aE^	79.36 ^aE^
Agar-agar	100 ^bA^	0 ^bB^	0 ^bB^	0 ^bB^	0 ^bB^	0 ^bB^
Apple pectin	100 ^bA^	100 ^cA^	100 ^cA^	50 ^cB^	50 ^cB^	50 ^cB^
Bovine gelatine	90.36 ^cA^	51.80 ^dB^	31.57 ^dC^	23.87 ^dD^	19.77 ^dE^	11.01 ^dF^
Carrageenan	n.d.	n.d.	n.d.	n.d.	n.d.	n.d.
Citrus pectin	66.67^dA^	66.67 ^eA^	66.67 ^eA^	66.67 ^eA^	66.67 ^eA^	66.67 ^eA^
CMC	n.d.	n.d.	n.d.	n.d.	n.d.	n.d.
Gum arabic	90.46 ^cA^	82.37 ^fB^	66.77 ^eC^	46.40 ^fD^	28.69 ^fE^	22.25 ^fF^
Karaya gum	97.62 ^aA^	85.79 ^gB^	75.87 ^fC^	75.87 ^gC^	63.1 ^gD^	48.73 ^gE^
Sodium alginate	100 ^bA^	100 ^cA^	100 ^cA^	50 ^cB^	25 ^hC^	12.5 ^hD^
Wheat starch	n.d.	n.d.	n.d.	n.d.	n.d.	n.d.
Xanthan gum	n.d.	n.d.	n.d.	n.d.	n.d.	n.d.

Each value is expressed as mean (n = 3) of triplicate analysis with standard deviation < 1. n.d.: Not determined since foaming capacity of the hydrocolloid is 0. ^a–h^ Means followed by different superscript lowercase letters indicate significant differences (*p* < 0.05) within column by Tukey’s test. ^A–F^ Means followed by different superscript uppercase letters indicate significant differences (*p* < 0.05) within row by Tukey’s test.

### 2.4. Functional Groups and Degree of Esterification

**Figure 4:**
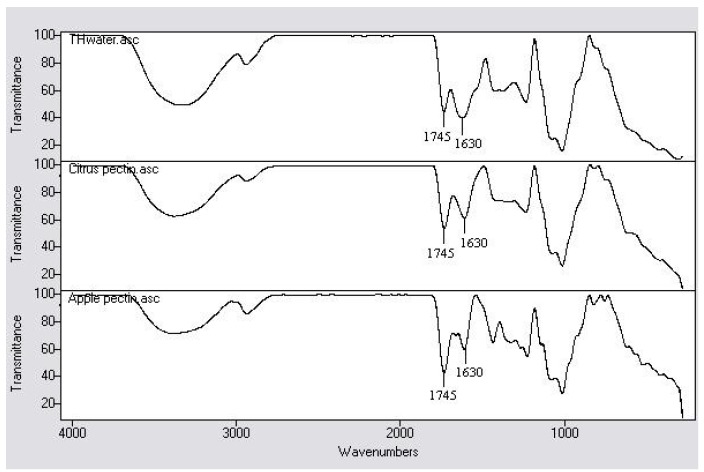
FT-IR spectra of tamarillo hydrocolloid (TH_water_), citrus and apple pectins.

The Fourier Transform Infrared (FT-IR) spectra of TH_water_ and hydrocolloids that exhibited similar patterns are presented in Figure 4. A moderately intense absorption pattern between 1,300 and 800 cm^‑1^ shows the ‘fingerprint’ region which is specific to each hydrocolloid. Based on this ‘fingerprint’ region, citrus and apple pectins were shortlisted from other commercial hydrocolloids that were compared (data not shown). It can be observed that TH_water_ had a very close ‘fingerprint’ region when compared with citrus pectin.

However, there was a clear difference that can be noted at bands at 1,745 and 1,630 cm^−1^. Citrus and apple pectins showed stronger absorptions at 1,745 cm^−1^ and weaker absorptions at 1,630 cm^−1^, whereas it was the reverse for TH_water_. The absorption bands at 1,745 and 1,630 cm^−1^ correspond to the C=O stretch of methyl esterified carbonyls and COO^−^ asymmetric stretching of carboxylate anions, respectively. A weaker absorption at 1,745 cm^−1^ (ester carbonyl groups) coupled with a stronger absorption at 1,630 cm^−1^ (carboxylate groups) as demonstrated by TH_water_ suggest that TH_water_ might be a low methoxyl pectin. On contrary, the relative proportions of the absorption bands at 1,745 and 1,630 cm^−1^ as presented by citrus and apple pectins were indicative of high methoxyl pectins [23]. Common absorption bands observed were at 3,400 cm^−1^ which is attributed to O-H stretching vibration, 2,930 cm^−1^ band due to C-H stretching of CH_2_ groups and 1,400–1,435 cm^−1^ band that corresponded to COO^−^ symmetric stretch of carboxylate anion [24,25].

These observations led to the quantitative measurement of degree of esterification (% DE) of TH_water_, citrus and apple pectins (Table 5) as described in Section 3.6. Citrus and apple pectins can be categorised in the same group since their DE were almost similar although there was a slight significant difference. Citrus and apple pectins studied in this work were high methoxyl pectins since their DE was higher than 50% [26] which correlates well with the stronger absorption observed at 1,745 cm^−1^ and weaker absorption at 1,630 cm^−1^. Conversely, the absorption bands at 1,745 and 1,630 cm^−1^ as showed by TH_water_ agreed well with the DE derived from the FT-IR spectrum. Based on these findings, it can be predicted that TH_water_ is a low methoxyl pectin since its DE was lower than 50% [26]. This propose the possible application of TH_water_ in reduced sugar food products and milk gels and desserts which have organic calcium ions since the gelation of low methoxyl pectin is governed by the interaction between pectin and calcium ions [27].

**Table 5:** Degree of esterification (DE) of tamarillo hydrocolloid (TH_water_), apple and citrus pectins measured using FT-IR spectroscopy.

Hydrocolloid	Degree of esterification (%)
TH_water_	49.47 ^a^ ± 0.23
Citrus pectin	68.00 ^b^ ± 0.19
Apple pectin	69.64 ^c^ ± 0.26

Each value is expressed as mean ± standard deviation (n = 3) of triplicate analysis; ^a–c^ Means followed by different superscripts indicate significant differences (*p* < 0.05) by Tukey’s test.

### 2.5. Monosaccharide Composition of Tamarillo Hydrocolloid

**Figure 5:**
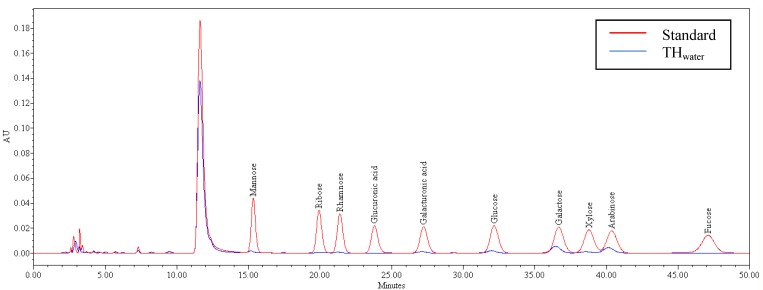
Chromatogram of PMP derivatives of monosaccharide standard and tamarillo hydrocolloid (TH_water_).

High resolution simultaneous separation of 10 derivatised monosaccharides using RP-HPLC is shown in Figure 5. TH_water_ was predominantly constituted of galacturonic acid (GalU), glucose (Glc), galactose (Gal) and arabinose (Ara) in the order Gal > Ara > Glc > GalU (Table 6).

Other neutral sugars such as mannose, ribose, rhamnose and xylose were found in trace quantities. Glucuronic acid and fucose were absent in TH_water_. Four major constituents found in TH_water_ were in close agreement to that of longan pulp. The polysaccharide purified from longan pulp using water extraction method was composed of 33.7% Gal, 32.8% Ara, 17.6% Glc and 15.9% GalU [28]. In addition, the monosaccharide profile of polysaccharide extracted from cupuassu pulp using water extraction method [16] was almost similar to that presented by TH_water_ and longan pulp.

**Table 6:** Monosaccharide composition (mol %) of hydrocolloid extracted from tamarillo puree (TH_water_).

Monosaccharide	Composition (mol%) ^a^
Mannose	Tr ^b^
Ribose	tr
Rhamnose	tr
Glucuronic acid	0
Galacturonic acid	0.98 ± 0.28
Glucose	7.05 ± 0.21
Galactose	51.63 ± 0.97
Xylose	tr
Arabinose	38.80 ± 0.69
Fucose	0

Each value is expressed as mean ± standard deviation (n = 3) of triplicate analysis. ^a^ mol% = (amount of individual monosaccharide/total amount of monosaccharide present in the sample) × 100; ^b^ tr: Traces < 0.01 mol%.

The consistency of these findings suggest the high probability that the aforementioned four common monosaccharides origining from different polysaccharides are similarly fractionated when a water extraction method is used. In contrast, the presence of mannose and xylose in trace quantities might be due to poor extractability of mannose- and xylose-containing polysaccharides using the water extraction procedure. The poor extractability might be explained by the fact that xylose and mannose, being the major constituents of hemicelluloses, were extremely stable to the water extraction method. This is due to the strong bonds between cellulose and hemicellulose that cause difficulties in cleavage, even by treatment with strong acids [29]. However, hemicellulose constituents (xylose and mannose) were more extractable when strong alkali (2 M or 4 M NaOH) were used [30]. Therefore, the extraction method plays a crucial role in achieving the right targeted monosaccharide profile that influences the functional properties of the extracted hydrocolloid. 

From the monosaccharide profile, functional groups and DE findings, TH_water_ shows a resemblance to pectic polysaccharide.

## 3. Experimental

### 3.1. Materials

For each batch of analysis, fresh tamarillos with maturity stage between 21–24 weeks were purchased from five different growers in the Cameron Highlands, Malaysia. To get a representative sample, fruits purchased from different growers were well mixed and selected for uniformity of ripeness, size, skin colour and free of defects. Other fruits (apple, mandarin orange, red dragon fruit, kiwifruit, marian plum, garden tomato, guava, water apple, jackfruit, pineapple, honeydew, mango, papaya and sapodilla) for screening purpose were purchased from fruit stalls in Selangor, Malaysia.

Undenatured ethanol (95%) was purchased from HmbG Chemicals (Hamburg, Germany). All reagents used for proximate analysis were of analytical grade. Agar-agar, bovine gelatine, carboxymethyl cellulose sodium salt (CMC), carrageenan, gum arabic, sodium alginate and xanthan gum were purchased from R&M Chemicals (Essex, UK). Gum karaya and pectin from citrus peel (galacturonic acid ≥ 74.0%) were purchased from Sigma-Aldrich (St. Louis, MO, USA and Copenhagen, Denmark), respectively. Starch from wheat and pectin from apples (degree of esterification, 70–75%) were purchased from Fluka (Steinheim, Germany and Buchs, Switzerland), respectively. Monosaccharide standards and HPLC grade chemicals used for monosaccharide profiling analysis were purchased from Sigma-Aldrich and Merck (Darmstadt, Germany), respectively.

### 3.2. Sample Preparation for Hydrocolloid Extraction

**Figure 6:**
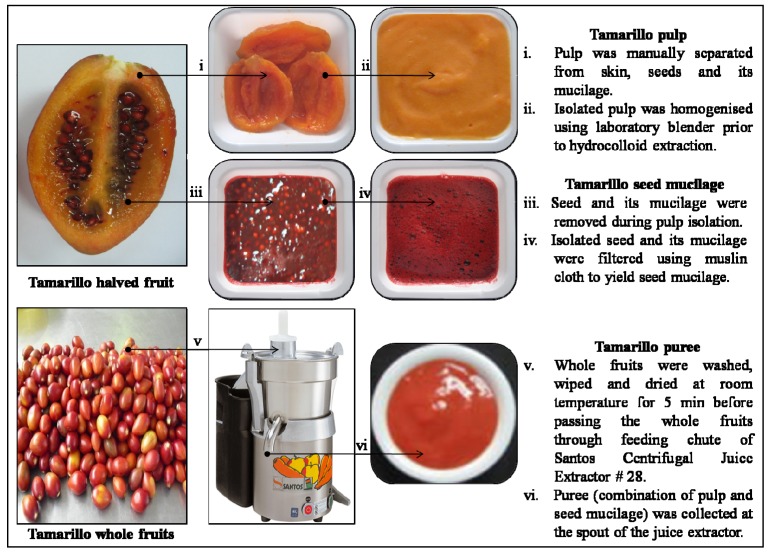
Preparation of pulp, seed mucilage and puree fractions from fresh Tamarillo fruits.

Edible fractions of 13 fresh fruits and orange peel were homogenised with increasing speed repeatedly (4 × 1 min) using a Waring laboratory blender (MS 153-5, Torrington, CT, USA). Although orange peel (raw material for commercial citrus pectin) is non edible, it was used for comparison study in hydrocolloid yield screening. Preparation of three fractions (pulp, seed mucilage and puree) of fresh tamarillo fruits was as described in Figure 6. Samples were packed in aluminium pouches and stored frozen (−18 °C) until analysis.

### 3.3. Screening of Various Fruits for High Hydrocolloid Yield

Fifteen tropical and subtropical fruits were screened for high hydrocolloid yield using the water extraction method as recommended by Yuliarti *et al*. [18] with some modifications. Homogenised fresh sample was extracted using distilled water at 50 °C for 30 min using 1:4 (w:v) sample to water ratio in shaking water bath. Extraction conditions were set based on optimum conditions described by Yuliarti *et al*. [18] and preliminary studies. The extract was centrifuged (3,300 g, 20 min) and the supernatant was decanted. Water at 50 °C was added to the pellet in the ratio of 1:1 (w:v) to extract any remaining hydrocolloid, and centrifuged again as before. 

Undenatured ethanol (95%) was added to the combined supernatants, the mixture was stirred and kept at 4 °C for 4 h to facilitate hydrocolloid precipitation. The mixture was centrifuged (3,300 g, 10 min) and the supernatant was discarded. The pellet was washed twice with 95% undenatured ethanol (1:1; w:v) and centrifuged again. The final pellet was placed on petri dish and oven-dried (Memmert, Schwabach, Germany) at 50 °C for 12 h until a constant weight was achieved. The dried pellet was then weighed for hydrocolloid yield calculation. Pulverisation of dried hydrocolloid using a pulverisette 14 variable speed rotor mill (Fritsch GmbH, Oberstein, Germany) was performed only for the fruit fractions that demonstrated highest yield and good feasibility of study. The selected dried hydrocolloid was passed through a 200 μm sieve (Fritsch GmbH), packed and stored at cool and dry conditions until further characterisation. 

Hydrocolloid yield on fresh (Y_f_) and dry (Y) weight basis were calculated using equations (1) and (2), respectively: 


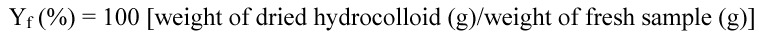



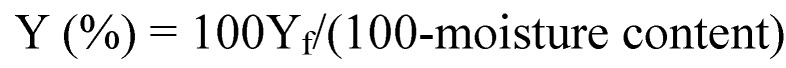


Since Y allows comparison with that of reported in literatures, the moisture content of fruits to be screened was determined prior to Y calculation. Fruit fraction that demonstrated the highest Y was used for the subsequent studies and thus tamarillo puree was selected. Water extracted, dried and pulverised tamarillo hydrocolloid is identified as TH_water_ thereafter.

Moisture content and dry matter were determined according to AOAC Method 934.01 [31]. Ash was analysed gravimetrically by incineration of samples in a muffle furnace for 2 h at 900 °C. The protein content estimated as % nitrogen × 6.25 (conversion factor) was determined with the Kjeldahl method on a Kjeltec^TM^ 8400 Analyzer unit (FOSS-Tecator AB, Hoganas, Sweden) which is consistent with AOAC method 978.04 [31]. Total starch content was determined using Megazyme Total Starch Assay procedure which is based on AOAC Method 996.11 [31]. Dietary fibre was calculated by difference using the following equation:


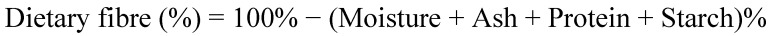


### 3.5. Functional Characterisation of Tamarillo Hydrocolloid in Comparison to that of Commercial Hydrocolloids

#### 3.5.1. Water-holding Capacity (WHC) and Oil-holding Capacity (OHC)

WHC and OHC were determined according to Robertson *et al*. [32] with some modifications. Distilled water or commercial olive oil (25 mL) were added to 250 mg of dry sample, stirred and left at room temperature for 1 h. After centrifugation, the residue was weighed. The WHC was expressed as g of water held per g of sample, while the OHC was expressed as g of oil held per g of sample.

#### 3.5.2. Emulsifying Activity (EA) and Emulsion Stability (ES)

EA and ES were evaluated according to Chau *et al*. [33] with some modifications. A DIAX 900 Heidolph homogeniser (Schwabach, Germany) was used to homogenise a 2% (w/v) sample suspension in water at 11,000 rpm for 30 s. Sunflower oil (100 mL) was then added and homogenised for another 1 min. The emulsions were centrifuged in 15 mL graduated centrifuge tubes at 1,200 g for 5 min, and the volume of the emulsion left was measured. To determine the ES, emulsions prepared by the above procedures were heated at 80 °C for 30 min in an oven, cooled to room temperature, and centrifuged at 1,200 g for 5 min. EA and ES were calculated using the following equations:


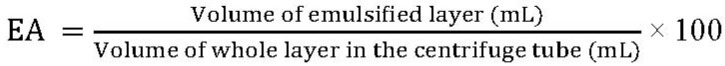



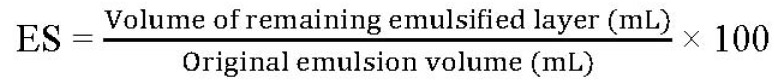


#### 3.5.3. Foaming capacity (FC) and Foaming Stability (FS)

FC and FS were determined according to the method described by Coffman and Garcia [34]. One gram of sample was mixed with 100 mL of distilled water and the suspension was homogenised vigorously at 13,000 rpm for 5 min using a DIAX 900 Heidolph homogeniser (Schwabach, Germany). The homogenised suspension was immediately transferred to a 250 mL graduated cylinder and the foam volume was measured. Foam volume changes were recorded at intervals of 1, 10, 30, 60, 90 and 120 min. FC and FS were calculated using the following equations:


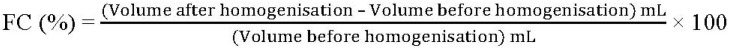



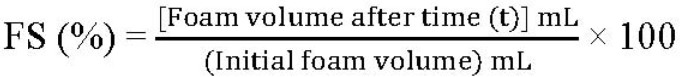


### 3.6. Functional Groups and Degree of Esterification Determination Using FT-IR Spectroscopy

Samples were redried and stored in desiccators prior to FT-IR analysis to avoid shifts in the spectra because of interference from moisture particles. FT-IR spectra were recorded using a universal ATR accessory with diamond/KRS-5 as the contact crystal on a Perkin Elmer Spectrum 100 FT-IR spectrophotometer with CsI detector (Shelton, CT, USA). The spectra were recorded at the absorbance mode from 4,000 to 400 cm^−1^ (mid infrared region) at the resolution of 4 cm^−1^ and 128 scans were co-added to obtain a high signal-to-noise ratio. At least duplicate spectra readings for each sample were performed. Degree of esterification (DE) was measured for pectin and pectin-like samples. Since DE is defined as (number of esterified carboxylic groups/number of total carboxylic groups) × 100, it is inferred that the ratio of the area of the band at 1,745 cm^−1^ (corresponding to the number of esterified carboxylic groups) over the sum of the areas of the bands at 1,745 cm^−1^ and 1,630 cm^−1^ (corresponding to the number of total carboxylic groups) should be proportional to the DE, *i.e.*, DE = A1745/(A1745 + A1630) [24,25]. The areas of interest were measured using OMNIC software version 8.0 (Thermo Nicolet Corp., Madison, WI, USA).

### 3.7. Monosaccharide Profiling Using HPLC

Monosaccharide standard solution was prepared as described by Lv *et al*. [35]. TH_water_ was hydrolysed to monomers with trifluoroacetic acid (TFA) prior to derivatisation with 1-phenyl-3-methyl-5-pyrazolone (PMP). The hydrolysis procedure described by Dai *et al*. [36] was carried out with a modification whereby the volume of all chemicals and sample solution needed for hydrolysis process was increased by 100×, *i.e.*, 10 mL was used instead of 100 μL. Derivatisation of monosaccharides with PMP [36] was done with the aforementioned modification until the step neutralisation with 0.3 M hydrochloric acid. Then, 300 μL of the resultant solution was evaporated to dryness using rotary evaporator and proceeded with liquid-liquid separation method [36].

Ten monosaccharides (glucose, galactose, arabinose, xylose, mannose, rhamnose, fucose, ribose, glucuronic acid and galacturonic acid) were separated using ZORBAX Eclipse Plus-C18 HPLC column (250 mm length, 4.6 mm internal diameter and 5 μm particle size, Agilent Technologies, Santa Clara, CA, USA). A Waters e2695 HPLC equipped with a Waters 2489 UV/Vis detector was used. The HPLC conditions and mobile phase composition were as described by Dai *et al*. [36]. The data were collected using an Empower2 software (Waters, Milford, MA, USA). The concentration of monosaccharides identified in TH_water_ were calculated by comparing their integration values of peak area to a calibrated standard curve.

### 3.8. Statistical Analysis

The statistical model was a one-way analysis of variance (ANOVA) for comparison of moisture content, extraction yield, WHC, OHC, EA, ES, FC and DE means. Twelve one-way ANOVA were performed to evaluate the significant differences between FS of different hydrocolloids. Six one-way ANOVA were carried out to determine the effect of time on FS of each hydrocolloid. Statistical analyses were carried out using Statistical Package for Social Science for Window, version 19.0 (SPSS Institute Inc., Cary, NC, USA). Treatment means were considered significantly different at *p* < 0.05 and whenever applicable, means were separated by Tukey’s HSD post hoc test.

## 4. Conclusions

Screening of various tropical and subtropical fruits for high hydrocolloid yield revealed that tamarillo can be a potential source of food hydrocolloid. A water extraction method was chosen since the method is safe for consumers and feasible for industrial scale-up. The hydrocolloid extracted from tamarillo puree consisted of 66.48% dietary fibre, 21.18% protein and 0.83% starch. The degree of esterification derived from FT-IR spectrum was 49.47%. The monosaccharides identified were mannose, ribose, rhamnose, galacturonic acid, glucose, galactose, xylose and arabinose. Good oil-holding capacity (2.11g oil/g dry sample), emulsifying activity (84.74%) and emulsion stability (94%) propose its possible application as a plant based food emulsifier. Besides that, it demonstrated a foaming capacity of 32.19% which is unique for a plant derived hydrocolloid. Interestingly, it showed better foam stabilisation (79.36% of initial foam volume) characteristics than bovine gelatine (11.01%) through 2 hours from the formation of the foam. These findings suggest that water extracted tamarillo hydrocolloid can be utilised as a low methoxyl pectin in reduced sugar products, milk gels and desserts. Ongoing works include extraction using different methods, and rheological and structural characterisations of other hydrocolloids from tamarillo to evaluate their potential as functional ingredients in food products.
